# Quantifying the Lack of Scientific Interest in Neglected Tropical Diseases

**DOI:** 10.1371/journal.pntd.0000576

**Published:** 2010-01-26

**Authors:** Dieter Vanderelst, Niko Speybroeck

**Affiliations:** 1 Faculty of Applied Economics, University of Antwerp, Antwerp, Belgium; 2 Institute of Tropical Medicine, Antwerp, Belgium; 3 Institut de recherche santé et société, Université Catholique de Louvain, Brussels, Belgium; New York Blood Center, United States of America

Since 1990 the World Health Organization uses the disability-adjusted life year (DALY) statistic to quantify the burden of diseases [1]. This indicator quantifies in one measure both the morbidity and the mortality due to disease. Estimating DALYs is intrinsically problematic since for some conditions only limited data are available [1],[2]. For several tropical diseases, especially those affecting people in the poorest countries, it has been argued that DALYs are systematically underestimated [1]–[3]. Because it is considered economically unprofitable, virtually no new drugs are being developed for this group of conditions [4],[5]. Being underestimated and lacking targeted drug development programs, these conditions have been termed neglected tropical diseases (NTDs). The list of infections that are considered to be NTDs varies depending on the author(s). However, they are usually taken to include those listed in Table 1 together with dracunculiasis and Buruli ulcer.

**Table 1:** The NTDs and the matched conditions included in the study.

Neglected Tropical Disease	DALY, WHO 2004	DALY, Hotez et al. 2006 [10]	Matched Condition	DALY, WHO 2004
Leprosy	194	200	Poliomyelitis	34
Onchocerciasis	388	500	Diphtheria	173
Chagas disease	430	700	Periodontal disease	320
Dengue	670	N/A	Appendicitis	418
Japanese encephalitis	681	N/A	Vitamin A deficiency	629
Trichuriasis	1,012	6,400	Hepatitis C	954
Hookworm disease	1,091	22,100	Bladder cancer	1,451
Trachoma	1,334	2,300	Otitis media	1,488
Trypanosomiasis	1,673	1,500	Multiple sclerosis	1,527
Schistosomiasis	1,707	4,500	Parkinson disease	1,710
Ascariasis	1,851	10,500	Ovary cancer	1,745
Leishmaniasis	1,974	2,100	Hepatitis B	2,067
Filariasis	5,940	5,800	Tetanus	5,283
**Mean**	**1,457**	**5,145**	**Mean**	**1,369**

The reported DALYs were taken from WHO 2004 estimates [7]. Estimated DALYs for the NTDs by Hotez et al. [10] are also included for comparison (see text for details). The listed DALYs have all been scaled by a factor 1/1,000.

Although there may be room for improvement in the calculation of DALYs related to NTDs, governments and policy makers use them to determine priorities in prevention and health care and therefore they cannot be ignored. Following Swingler et al. [6], research efforts targeted at a disease should ideally be in proportion to its global health impact. However, NTDs are prone to be less considered by the scientific community than what their DALYs would call for. Therefore, it appeared worthwhile to investigate whether NTDs are not neglected twice: once by being attributed an underestimated DALY and again by limited scientific attention.

## Indicators of Scientific Interest

We did not include all diseases that are considered NTDs in our analysis, because only 13 of them are listed in the DALY estimates published by WHO [7]. For these 13 NTDs, 13 other non-NTD conditions with matched estimated DALYs (Table 1) were selected for searches of the scientific literature. The matched diseases were chosen on the basis of two criteria: (1) the disease had to be listed as a separate condition in the DALY estimates and (2) we chose the condition with the closest matching DALY. The DALYs associated with each group of conditions did not differ significantly based on a paired Wilcoxon signed rank test and a paired *t*-test after log transformation of the DALYs (in both cases, 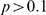).

We queried both the freely available PubMed database and the ISI Web of Science database to determine the number of publications for each disease per year since 1970. These two databases complement each other and using both of them is likely to give a more balanced image of research efforts. The Web of Science is more extensive, listing publications from many fields of science, and contains over 85 million records. PubMed contains fewer records (about 19 million) but has better coverage of older publications [8]. In both databases the disease name as listed in Table 1 was entered as a search term.

**Figure 1:**
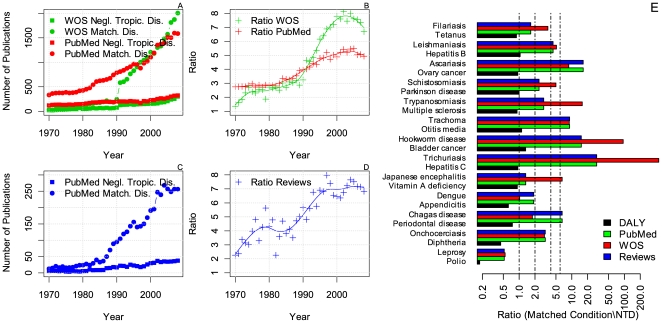
Number of publications on NTDs and matched conditions. (A) The number of publications in PubMed and Web of Science as a function of time for the NTDs and the matched conditions. (B) The ratio between the number of publications in PubMed and Web of science on NTDs and the matched conditions as a function of time. The lines are spline smoothed versions of the ratio data. (C, D) Idem as (A, B) but for the reviews found in the PubMed database. (E) The ratios of the estimated DALYs and number of publications for each NTD-matched condition pair summed across time. The vertical lines indicate a ratio of 1, 2, 4 and 6 respectively. Notice that the x-axis has a logarithmic scaling.

Our analysis shows that NTDs are less researched than the matched conditions with comparable DALYs (Figure 1). Moreover, the gap has widened during recent years. Averaged across time, the number of papers on the matched conditions was four times higher in the PubMed database and six times higher in Web of Science. However, around 2003–2004, when the discrepancy between the number of papers published for each group of conditions was the largest, the number of papers published on NTDs was five (PubMed) to eight (Web of Science) times lower than on conditions with similar estimated impact.

In line with these findings, the number of reviews in PubMed on NTDs was lower than for the matched diseases (Figure 1). The number of reviews on a condition is considered important for clinical practice [6]. Previously it was found that the number of systematic reviews on diseases is moderately correlated with their assigned DALYs and that this correlation holds for both the established market economies as well as for the global disease impact [6]. Contradicting this observation, we found that the number of reviews in PubMed on NTDs was much lower than for the matched diseases. Therefore, focusing on the neglected diseases and matched counterparts reveals a discrepancy that has gone unnoticed by aggregating many diseases.

Although there was considerable variance between the disease pairs, for all but one NTD-matched condition pair the matched disease attracted more research. This did not hold for polio and leprosy (see Figure 1D). More papers were published on leprosy than on polio in the period 1970–2009. We accounted for this in two ways. First, polio and leprosy constitute the worst matched disease pair. The estimated impact of leprosy is more than five times higher than that of polio. However, no better matching disease was available for leprosy. Second, poliomeylitis has been eradicated in many developed areas in the world, which might reduce the incentive for carrying out research on this topic.

The difference in scientific output between NTDs and the matched conditions is higher for the Web of Science than for PubMed. This is due to both a lower number of publications on NTDs and a slightly higher number of publications on the matched conditions in this database. The Web of Science lists only research published in well-established journals. It is known that research on NTDs is less likely to be featured in such journals [9]. This stresses the importance of targeted journals on NTDs such as *PLoS Neglected Tropical Diseases*.

The disproportionally low research interest in NTDs is doubly worrying if one considers that the DALYs associated with NTDs are generally assumed to be underestimated. For example, Hotez et al. [10] list updated estimates for the DALYs associated with several NTDs (see also Table 1). These updated estimates clearly indicate that their real impact on health and quality of life worldwide may actually be considerably higher than that of the matched conditions.

## Indicators of Public Interest

The Google internet search engine provides access to the search volume for terms, using their freely available Google Trends application. For each disease name we extracted the proportion of queries processed by Google from 2004 to present. The number of Web pages found by Yahoo on NTDs and the matched conditions was also retrieved.

We also found that on the internet less information about NTDs is available to the public. Additionally, this information is also accessed less often. Indeed, the number of internet searches processed by Google in the period 2004–2009 was 2.25 times lower for NTDs than for the matched conditions. Similarly, the number of Web pages found by Yahoo on NTDs was lower than for the matched conditions (Wilcoxon signed rank test, 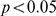). This indicates that the lack in research interest in NTDs is sustained by a lack in public interest as well.

## Changing Tides?

There are preliminary indications that there now is an increased interest in NTDs. The use of the term “neglected tropical disease” has, across both databases, risen monotonically since 2004 (the first item we found on the topic was an editorial by Holland in 1991 [11], which appeared in the Web of Science). The number of records mentioning neglected tropical diseases has risen from one in PubMed and four in the Web of Science in 2005 to 32 and 69, respectively, in 2008. This increase is mostly due to articles published in *PLoS Neglected Tropical Diseases*. An increase in internet queries on NTDs was observed as well. In 2004, matched diseases were queried 3.3 times as often as NTDs. In the first half of 2009 this ratio dropped to 1.8.

Moreover, the ratio between the number of publications of the two groups of diseases reached its peak in 2003–2004. Since then, the ratio seems to be falling. This finding is in concordance with the recent increase in research effort targeted at drug development for NTDs [12]. Indeed, new international initiatives may further contribute significantly toward reducing the so-called 10/90 gap. The 10/90 gap concept refers to the finding that only 10% or less of the global expenditure on medical research and development is directed toward neglected health problems [13].

The recent increase in academic and public interest in NTDs are important indicators of change. These and other indications of a turning tide need to be confirmed in the future. It should also be noted that, even when research effort into NTDs would, in a few years from now, match that of diseases with equal impact, there still is a need to pay off the arrears of the past (see Figure 1D). Similarly, although it has been argued that the increase in the number of drug development programs is a step in the right direction, the efforts are still too small to change the situation profoundly [5]. Also, the current paucity of research on NTDs might slow down the readjustment of their DALYs.

More research is needed in order to gain a more realistic estimate of the burden of these diseases; the resulting higher estimates of DALYs would probably cause these diseases to attract more research. By the same token, a lack of attention to these diseases could be self-perpetuating. It will be necessary for civil society, scientists, and policymakers alike to break this cycle so that some of the most common infections among the 2.7 billion people living on less than US$ 2 per day [3], receive the attention they deserve.
